# Updating MISEV: Evolving the minimal requirements for studies of extracellular vesicles

**DOI:** 10.1002/jev2.12182

**Published:** 2021-12-25

**Authors:** Kenneth W Witwer, Deborah CI Goberdhan, Lorraine O'Driscoll, Clotilde Théry, Joshua A Welsh, Cherie Blenkiron, Edit I Buzás, Dolores Di Vizio, Uta Erdbrügger, Juan M Falcón‐Pérez, Qing‐Ling Fu, Andrew F Hill, Metka Lenassi, Jan Lötvall, Rienk Nieuwland, Takahiro Ochiya, Sophie Rome, Susmita Sahoo, Lei Zheng

**Affiliations:** ^1^ Departments of Molecular and Comparative Pathobiology and Neurology and The Richman Family Precision Medicine Center of Excellence in Alzheimer's Disease Johns Hopkins University School of Medicine Baltimore Maryland USA; ^2^ Department of Physiology Anatomy and Genetics University of Oxford Oxford UK; ^3^ School of Pharmacy and Pharmaceutical Sciences Panoz Institute and Trinity Biomedical Sciences Institute (TBSI) & Trinity St. James's Cancer Institute (TSJCI) Trinity College Dublin Dublin Ireland; ^4^ INSERM U932, Institut Curie PSL Research University Paris France; ^5^ Translational Nanobiology Section Laboratory of Pathology Center for Cancer Research National Cancer Institute National Institutes of Health Bethesda Maryland USA; ^6^ Department of Molecular Medicine and Pathology The University of Auckland Auckland New Zealand; ^7^ Department of Genetics Cell‐ and Immunobiology HCEMM‐SU Extracellular Vesicles Research Group, and ELKH‐SE Immune‐Proteogenomics Extracellular Vesicles Research Group Semmelweis University Budapest Hungary; ^8^ Department of Surgery Department of Pathology & Laboratory Medicine Samuel Oschin Comprehensive Cancer Institute Cedars‐Sinai Medical Center Division of Cancer Biology and Therapeutics Los Angeles California USA; ^9^ Department of Medicine Division of Nephrology University of Virginia Charlottesville Virginia USA; ^10^ Exosomes Laboratory & Metabolomics Platf CIC bioGUNE‐BRTA IKERBASQUE, CIBERehd Bilbao Spain; ^11^ Exosome Research and Translational Center The First Affiliated Hospital Otorhinolaryngology Hospital Sun Yat‐sen University Guangzhou China; ^12^ Department of Biochemistry and Genetics La Trobe Institute for Molecular Science La Trobe University Bundoora VIC Australia; ^13^ Institute of Biochemistry and Molecular Genetics Faculty of Medicine University of Ljubljana Ljubljana Slovenia; ^14^ Krefting Research Centre University of Gothenburg Göteborg Sweden; ^15^ Laboratory of Experimental Clinical Chemistry and Vesicle Observation Center Amsterdam University Medical Center University of Amsterdam Amsterdam The Netherlands; ^16^ Department of Molecular and Cellular Medicine Tokyo Medical University Tokyo Japan; ^17^ CarMeN Laboratory (INSERM 1060, INRAE 1397) University of Lyon & Faculty of Medicine Lyon‐Sud Pierre‐Bénite France; ^18^ Cardiovascular Research Institute Icahn School of Medicine at Mount Sinai New York New York USA; ^19^ Department of Laboratory Medicine Nanfang Hospital Southern Medical University Guangzhou China

**Keywords:** ectosomes, exosomes, extracellular vesicles, microvesicles, MISEV, reproducibility, rigor, standardization

## Abstract

The minimal information for studies of extracellular vesicles (EVs, MISEV) is a field‐consensus rigour initiative of the International Society for Extracellular Vesicles (ISEV). The last update to MISEV, MISEV2018, was informed by input from more than 400 scientists and made recommendations in the six broad topics of EV nomenclature, sample collection and pre‐processing, EV separation and concentration, characterization, functional studies, and reporting requirements/exceptions. To gather opinions on MISEV and ideas for new updates, the ISEV Board of Directors canvassed previous MISEV authors and society members. Here, we share conclusions that are relevant to the ongoing evolution of the MISEV initiative and other ISEV rigour and standardization efforts.

1

The minimal information for studies of extracellular vesicles (MISEV) is a major initiative towards fulfilling the mission of the International Society for Extracellular Vesicles (ISEV): advancing extracellular vesicle (EV) science globally. A first publication on minimal requirements, co‐authored by the ISEV Board in 2014 and now referred to as “MISEV2014” (Lotvall et al., 2014) was followed by a lengthier, more comprehensive document in 2018 (Théry et al., 2018). The “MISEV2018” product received input from more than 400 scientists, 382 of whom became co‐authors. MISEV2018 presented recommendations in six areas: EV nomenclature, sample collection and pre‐processing, EV separation and concentration, EV characterization, functional studies, and reporting requirements/exceptions. In preparing the first “MISEV2018” draft for detailed feedback from co‐authors, the ISEV Board was guided by prior input from the ISEV community, solicited through a survey in 2016 (Witwer et al., 2017). Among other outcomes, the survey reinforced that ISEV members wished to be more involved in preparing the guidelines and revealed key topics to address. Looking forward to a new iteration of MISEV, ISEV again sought community input to understand engagement with MISEV, determine how the guidelines can be improved, identify new topics that require attention, and define the relationship of MISEV with other rigour initiatives within or allied with ISEV. Here, we examine this latest crowdsourcing effort and what the results will mean for the next MISEV product and other ISEV initiatives.

## 2 SURVEY METHODS, RESPONSES, AND RESPONDENTS

A 26‐entry survey was prepared by the co‐coordinating authors of MISEV2018 and other members of the ISEV Board (Supplemental File 1), based in part on the pre‐MISEV2018 survey in 2016 (Witwer et al., 2017). Nineteen questions were mandatory, while seven entries could be answered optionally. The survey was sent by email invitation to 2768 addresses of individuals who were (1) ISEV members in any year from 2018 to 2020 and/or (2) MISEV2018 co‐authors. It was also sent via internet link to 20 ISEV Board members and adjuncts. The survey was open from November 1 to November 11, 2020, with one email reminder on November 8 to those who had not yet responded. Of the 2788 total invitations, 984 remained unopened, and approximately 100 bounced back or had opted out of emails from the survey system. However, more than 60% of recipients opened the email, and 764 answered all mandatory questions. Of these, 653 (85%) also responded to the majority of the optional questions. Respondents spent an average of 11 minutes each answering the survey. In comparison, the October 2016 questionnaire garnered 187 responses, and 139 (74.3%) also answered the majority of non‐mandatory responses.

**FIGURE 1:**
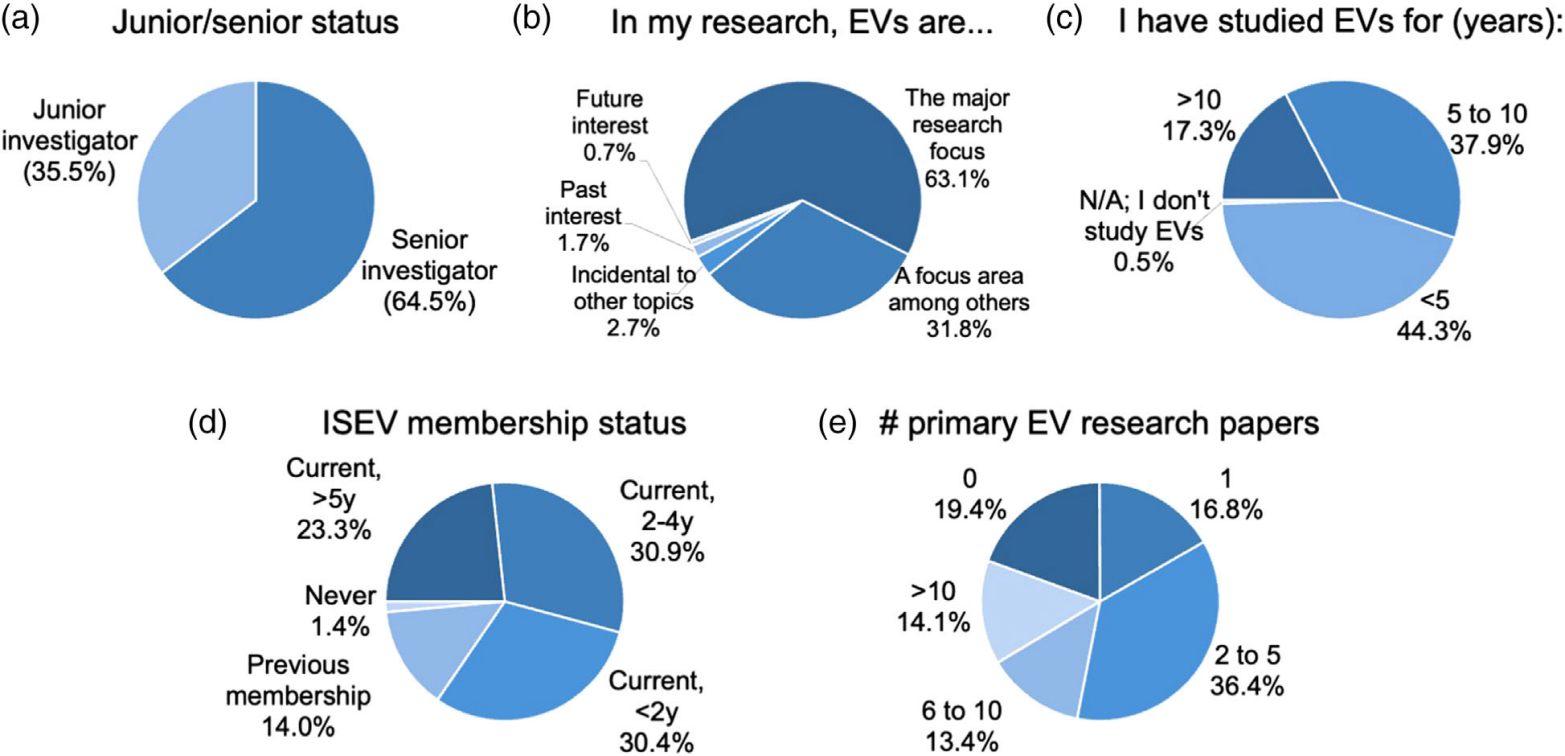
Status and EV experience of MISEV survey respondents. All questions were answered by all 764 respondents unless indicated in parentheses in the title of the respective panel

Survey respondents were mostly senior investigators, outnumbering junior investigators almost two‐to‐one (Figure 1a; ISEV defines “junior investigator” as an individual without a terminal degree, e.g., PhD, MD, DVM, or who received a terminal degree up to 4 years in the past). Almost 95% considered EVs to be their “major research focus” or a “large focus area” (Figure 1b), and a slight majority had studied EVs for five years or longer (Figure 1c). Almost 85% of respondents reported current ISEV membership, and >23% had been ISEV members for five or more years (Figure 1d). Eighty percent had co‐authored at least one primary research peer‐reviewed publication or preprint on EVs, and >14% reported more than 10 EV publications (Figure 1e).

**Conclusion**: Since most 2020 respondents are highly engaged with EV research and are contributing to the literature, an update to MISEV would draw on strong and diverse expertise.

## 3 ENGAGEMENT WITH MISEV

**FIGURE 2:**
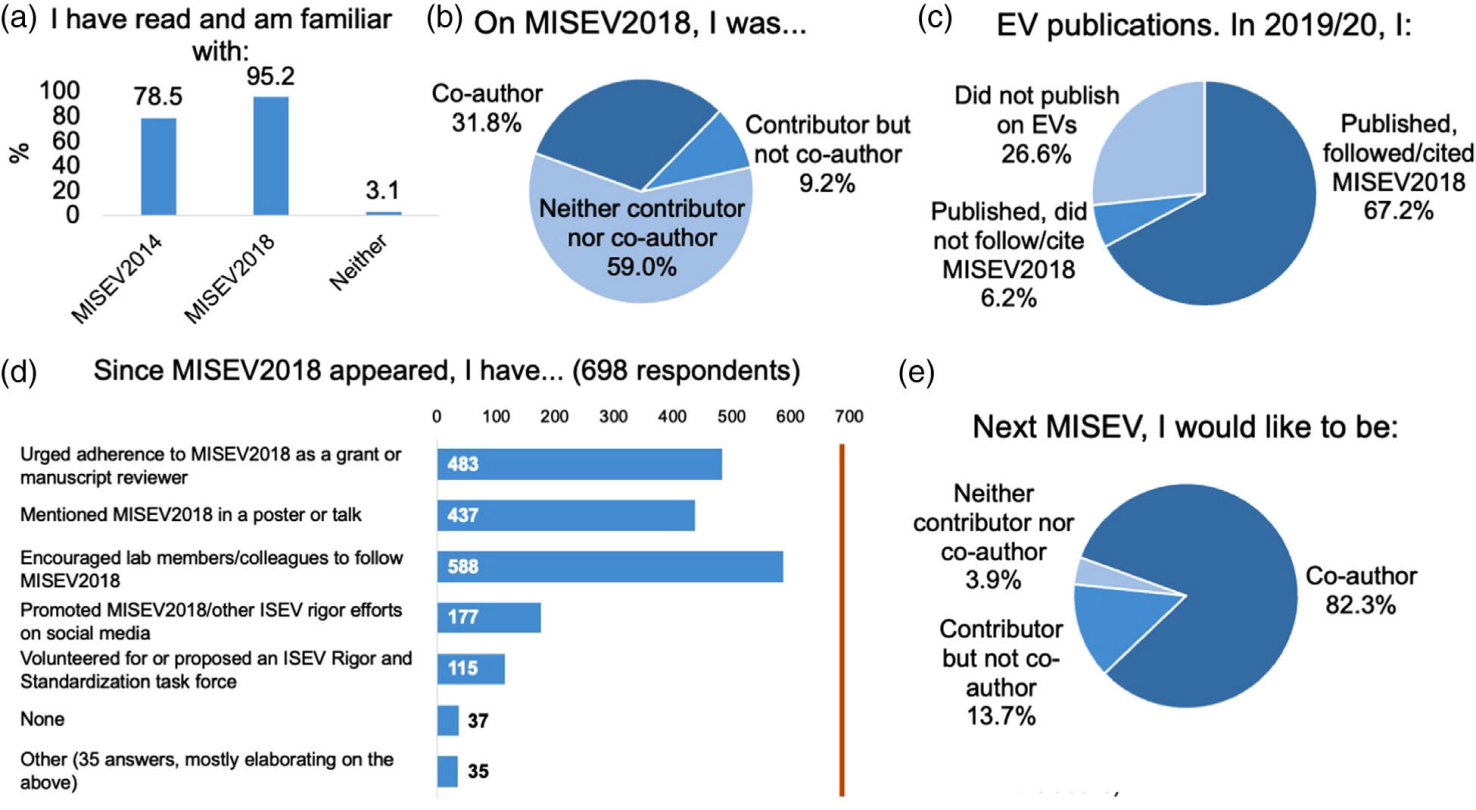
Respondent engagement with MISEV. All questions were answered by all 764 respondents unless indicated in parentheses in the title of the respective panel

Respondents were overwhelmingly familiar with MISEV2018, even though most were not co‐authors, and many had also engaged with MISEV in their published work and personal outreach. Most respondents were familiar with MISEV2018 (>95%) and MISEV2014 (around 79%; Figure 2a). However, the majority of respondents were not co‐authors of MISEV2018 (Figure 2b). Less than one‐third of respondents had been co‐authors of MISEV2018, although several others said they had contributed to MISEV2018 but were not co‐authors. Among published senior scientists, the majority were MISEV2018 co‐authors or contributors (not shown). Of 512 respondents who had published on EVs in 2019 or 2020, approximately 92% said that they had followed and/or cited MISEV2018 in their work, while about 8% had not (Figure 2c). Six hundred eighty‐eight individuals indicated how they had promoted MISEV2018 since its appearance (optional Question 9). Most free‐form “other” answers to this question (35) emphasized distinct aspects of the main answer choices or mentioned MISEV2018 promotion in publications and teaching activities (Figure 2d). For a possible new version of MISEV, 82% of respondents wished to contribute as co‐authors, and another 14% wished to contribute without an author byline. Only 4% did not want to be involved (Figure 2e).

**Conclusion**: There was strong overall engagement with MISEV among respondents, who represented an even larger pool of potential co‐authors than those involved in MISEV2018. However, approximately one‐third of respondents did not publish on EVs following the MISEV2018 publication or did not follow the guidelines.

## 4 VIEWS OF MISEV: RESPONDENTS

Respondents' views on MISEV were canvassed through a question identical to one in the 2016 survey, highlighting similarities and differences. In 2020, as in 2016, almost all respondents felt that minimal requirements are important: 96% in 2020 and 94% in 2016 (Table 1). However, “support” for the prevailing MISEV approach is substantially stronger, at 80% in 2020 compared with 53% in 2016. Whereas 25% considered MISEV “too restrictive” in 2016, only 11% felt this way in 2020; the view of MISEV as an “unnecessary imposition” fell from 3% to under 1%. Those who felt the guidelines are important but not strict enough also declined, from 16% in 2016 to 5% in 2020. “No strong feelings” held steady at approximately 3%.

**TABLE 1:** Views on MISEV: Respondents

Viewpoint on MISEV	2016 % (*n*)	2020 % (*n*)
Important/support	52.9 (73)	80.1 (559)
Important but too restrictive	25.4 (35)	11 (77)
Important but not strict enough	15.9 (22)	5.3 (37)
Unnecessary imposition	2.9 (4)	0.7 (5)
No strong feelings	2.9 (4)	2.9 (20)
**Total respondents**	691	138

**Conclusion**: Since the 2020 group of survey respondents is more comfortable with the degree of rigor of the MISEV guidelines than the 2016 respondents, a “hold the course” approach for the next MISEV update is likely more advisable than a major overhaul. Although the proportion of critical views has declined, these views (too restrictive/not strict enough) should still be addressed carefully in any MISEV revision.

## 5 VIEWS OF MISEV: RESPONDENTS' COLLEAGUES

Although more than a quarter of respondents had not received relevant feedback from colleagues about MISEV2018, most had (Table 2). In response to a question allowing multiple answers, 50% had heard colleagues endorsing MISEV2018, and 32% had heard other praise for the initiative. Complaints included suggestions that MISEV2018 was “too restrictive (25%),” ”too long(16%),” or had left out important topics or details (9%). Some had heard suggestions that MISEV2018 did not have enough uptake in the field (10%) or was not taken seriously (9%). Free‐form answers (“other”) mostly expanded on positive views, but some reported opinions that “gaming the system” was possible and that MISEV could be used unfairly during peer review.

**TABLE 2:** Views of MISEV: Feedback from respondents' colleagues (multiple answers permitted)

Feedback	%	*n* (of 698)
Endorsement of MISEV2018	50.1	350
Praise for MISEV2018	32.2	225
Complaints that MISEV2018 is too restrictive	24.8	173
Complaints that MISEV2018 is too long	15.9	111
Complaints that MISEV2018 left out important topics or details	9.3	65
Suggestions that MISEV2018 has not had wide enough uptake	9.9	69
Suggestions that MISEV2018 is not taken seriously	9.0	63
None of the above or have not received feedback	26.5	185

**Conclusion**: Despite many positive comments on MISEV, criticism of MISEV is clearly present in the community beyond the survey respondents. While maintaining rigor, future MISEV authors will want to address these views as much as possible, making clear that MISEV is a tool for advancing rigorous science, not for placing unreasonable barriers in front of newcomers to the field.

## 6 HAVE ISEV RIGOUR EFFORTS BEEN SUCCESSFUL?

Efforts to encourage rigour should consider assessment of impact, with the goal of guiding ongoing and future efforts. Citation metrics show solid uptake of MISEV2018, which has received, on average, more than four times as many citations per year as MISEV2014; however, the growth of the field must also be considered. More important than citations is whether MISEV is associated with quality or has promoted quality. Following MISEV2014, the initial EV‐TRACK publication reported that studies citing MISEV tended to have reporting of higher quality than those that did not (Van Deun et al., 2017). The ISEV Rigor and Standardization Subcommittee recently reported on trends in EV methods (Nieuwland et al., 2020; Royo et al., 2020) as an update to an earlier ISEV publication (Gardiner et al., 2016). Another assessment was published in 2021 by Poupardin et al. (2021). The authors found that EV publication quality, as measured by number of characterization methods and number of queried markers, had improved from 2012 to 2020, and that this improvement in quality was correlated with citation of MISEV (Poupardin et al., 2021).

MISEV survey takers were asked about their views on the overall quality of the EV literature and the quality of method reporting since MISEV2018 appeared. On overall publication quality, almost 71% said that it had improved since 2018 and credited ISEV efforts including MISEV with contributing (Table 3). An additional 3% suggested an improvement in quality, but independent of ISEV efforts. Seventeen percent felt that quality had stayed roughly the same, and just over 1% said that there was a decline in quality, perhaps because MISEV2018 had not been followed or had not achieved sufficient reach. Eight percent of respondents opted to give free‐form answers. On methods reporting specifically, there was an almost identical distribution of responses (Table 3). For both questions, most of the free‐form answers indicated no opinion, a lack of familiarity with MISEV, or that two years might not be enough time to assess changes in the field.

**TABLE 3:** Perceptions of efficacy of MISEV and other ISEV efforts

In the overall quality/method reporting of literature since MISEV 2018, there has been:	Overall quality, % (*n*)	Method reporting, % (*n*)
…an improvement, and MISEV2018/other ISEV efforts have contributed to this	70.6 (488)	70.4 (483)
…an improvement, but not because of MISEV2018/other ISEV efforts	2.7 (19)	4.4 (30)
…no clear improvement or decline compared with previous years, perhaps because MISEV2018 is ineffective or its positive influence is balanced by a massive influx of low‐quality studies	17.2 (119)	18.2 (125)
…a decline despite MISEV2018 (e.g., because MISEV2018 has not had sufficient uptake in the field or is known but not followed)	1.4 (10)	1.0 (7)
Other (free‐form response)	7.9 (55)	6.0 (41)
**Total respondents**	691	686

**Conclusion**: MISEV is viewed by respondents as contributing to overall quality and method reporting in publications, a view that is supported by assessments of the literature. However, as inexperienced researchers enter the field in large numbers, redoubled efforts may be needed to increase awareness of MISEV and other rigor initiatives.

## 7 MISEV UPDATE: LENGTH AND OVERALL FOCUS

Since MISEV2018 was a much longer article than MISEV2014, what length would be appropriate for the next MISEV? A plurality of respondents (38%) voted for “around the same length and covering the same topics, but with updated details and references” (Table 4). Another 19% agreed to a similar length, but “at least partially new topical focus.” However, another 19% envisioned a shorter MISEV: 3.7% simply preferring fewer details and/or topics in one document, but 15% suggesting that extensive details could be farmed out to accompanying guidelines articles (Table 4). In other words, a MISEV “constellation” around a central document. 18.5% called for a longer, more comprehensive MISEV (Table 4).

**TABLE 4:** Length and topical preferences for MISEV update

Preferred form of a MISEV update, compared with MISEV2018	% (*n*, of 655)
Longer and more comprehensive, incorporating more topics and details	18.5 (121)
Around the same length and covering the same topics, but with updated details and references	38.2 (250)
Around the same length, but with at least partially new topical focus	18.4 (127)
Shorter, with fewer details and/or topics	3.7 (24)
Shorter, with fewer details, but accompanied by additional, detailed guidelines articles on specific topics (like EV sources, methods)	15.0 (98)

**Conclusion**: Amongst some support for vastly differing approaches to a MISEV update, a similar length is preferred by a majority. However, many respondents appear to be open to new topical coverage and changes to the distribution of details across MISEV and possible accompanying documents.

## 8 MISEV UPDATE: PREVIOUS SECTION CONTENTS AND NEW EMPHASES

**FIGURE 3:**
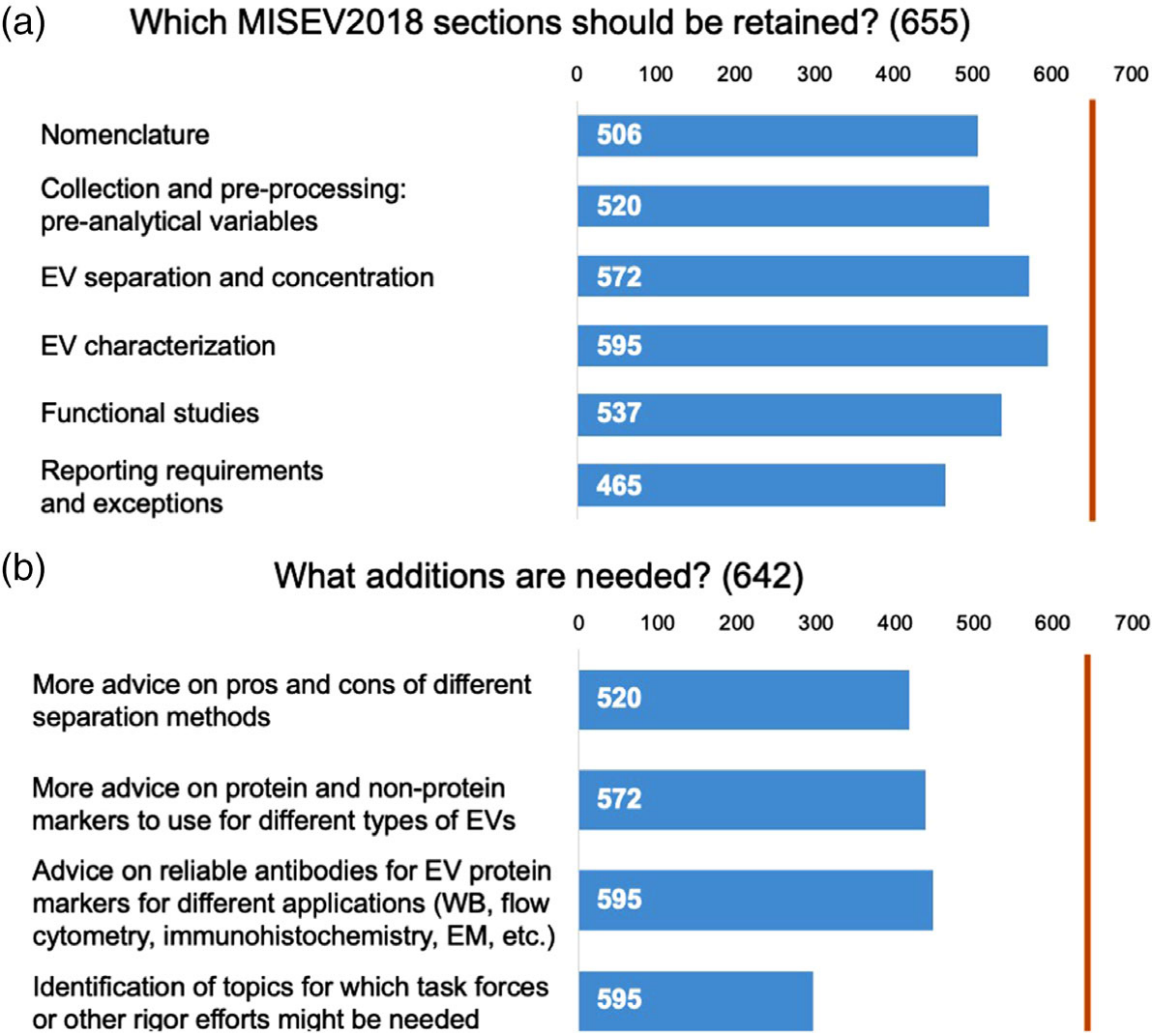
ISEV2018 sections to retain and topics to add. The total number of respondents for each question is indicated in parentheses in each panel title and by the vertical orange line; exact number of responses for each option is shown within each bar. WB, western blot; EM, electron microscopy

MISEV2018 had six main sections, and a majority of respondents voted to retain each (Figure 3a). At 71%, “Reporting requirements and exceptions” had the least support, while “EV Characterization” had the most, with 91%. Based on discussions during MISEV2018 preparation and informal feedback, respondents were asked to choose any number of four focus areas for inclusion in an update. Between 65% and 70% of responses to this optional question favoured addition of more information on separation methods, protein markers of EV subtypes, and antibody recommendations (Figure 3b). Forty‐six percent felt that potential topics for future task forces should be indicated (Figure 3b). One eighty‐nine free‐form answers were also given in response to the two content questions. After removing duplicated responses, comments indicating a lack of familiarity with MISEV (e.g., requesting a redundant section or content), and several helpful suggestions to focus on checklists and bullet‐points instead of lengthy text, the remaining topical input was sorted subjectively. Topics are shown in Table 5.

**TABLE 5:** Free‐form suggestions: Possible additions to MISEV

Topic	Comments (#)
Clinical applications of EVs, such as regulatory requirements, cGMP‐compliant production, quality control, and assessment of biomarker and therapeutics applications.	37
In vivo studies, including specific models; not including biodistribution	20
New developments in EV characterization	16
Non‐mammalian EVs, especially bacterial EVs; biosafety considerations due to enveloped viruses	15
Source‐specific considerations in EV studies; biofluid task forces	12
Considerations for EV labeling: types of labeling, pitfalls, controls, and post‐labeling purification	12
EV tracking, biodistribution, pharmacokinetics, and immune responses	12
Practical offerings: SOPs/movies of techniques/“case studies” of common issues and troubleshooting	11
EV stability, storage, and handling	9
Appropriate controls in EV studies; normalization and reference genes	8
Non‐EV extracellular particles/contamination/co‐isolates	8
EV subtypes, including their nomenclature	6
New development in EV separation methods	5
EV reference materials and standards	5
Obtaining EVs from specific cells, for example, neurons	5
Request for checklists and bullet‐points instead of lengthy text	5
Recommendation of specific antibodies	4
Considerations specific to single‐particle analysis	4
EV uptake	4
Issues of EV scale‐up and cell engineering	3
EVs as delivery vehicles; loading	3
EV biogenesis	3
Functional assays	3
Other: inter‐laboratory comparisons (1), EV history (1), measuring effects of cell stress (2)	4

**Conclusion**: The basic sections of MISEV2018 retain support. The newly suggested topics should be considered carefully for inclusion in the next MISEV or assigned to task forces (if this has not already been done) for more attention.

## 9 MISEV UPDATE: RELATIONSHIP OF MISEV AND RIGOUR AND STANDARDIZATION EFFORTS

In light of the formation of the Rigor and Standardization (R&S) subcommittee in 2019 and numerous topical task forces (Börger et al., 2020; Clayton et al., 2019; Erdbrügger et al., 2021; Nieuwland et al., 2020; Welsh, Van Der Pol, Arkesteijn, et al., 2020, Welsh, van der Pol, Bettin, et al., 2020), some respondents said that R&S efforts are complementary to MISEV and should be pursued as separate published products (34%); however, almost 40% felt that task force products could be integrated into a MISEV update (data not shown).

**Conclusion**: There is no consensus on how MISEV and R&S efforts should integrate. The R&S Subcommittee can thus proceed with an ad‐hoc approach to how topic‐specific guidelines are integrated into or related to MISEV. An update to MISEV may indeed review key findings or questions of the existing task forces.

## 10 MISEV UPDATE: WHAT ABOUT EV‐TRACK AND REPORTING REQUIREMENTS?

The EV‐TRACK knowledgebase (evtrack.org) allows scientists to report detailed methods of EV studies and identifies opportunities for improvement of reporting. EV‐TRACK was introduced by Van Deun et al. (2017) and has been endorsed by MISEV2018 and by the ISEV journals. Thirty‐seven percent of respondents to this survey endorsed EV‐TRACK and had used it themselves, 34% endorsed it but had not used it, and 28% were not familiar with EV‐TRACK or had no opinion. 2.7% did not support endorsement of EV‐TRACK and commented on why they did not. Most of these comments addressed technical issues with the EV‐TRACK website or encouraged changes to become or include a MISEV “checklist” application.

**Conclusion**: Despite majority endorsement of EV‐TRACK, many respondents had not used the site or were unfamiliar with it, suggesting opportunities for outreach. Updates to the knowledgebase might take comments into account.

## 11 MISEV UPDATE: WHO SHOULD BE AN AUTHOR? AND WHEN TO PUBLISH?

**FIGURE 4:**
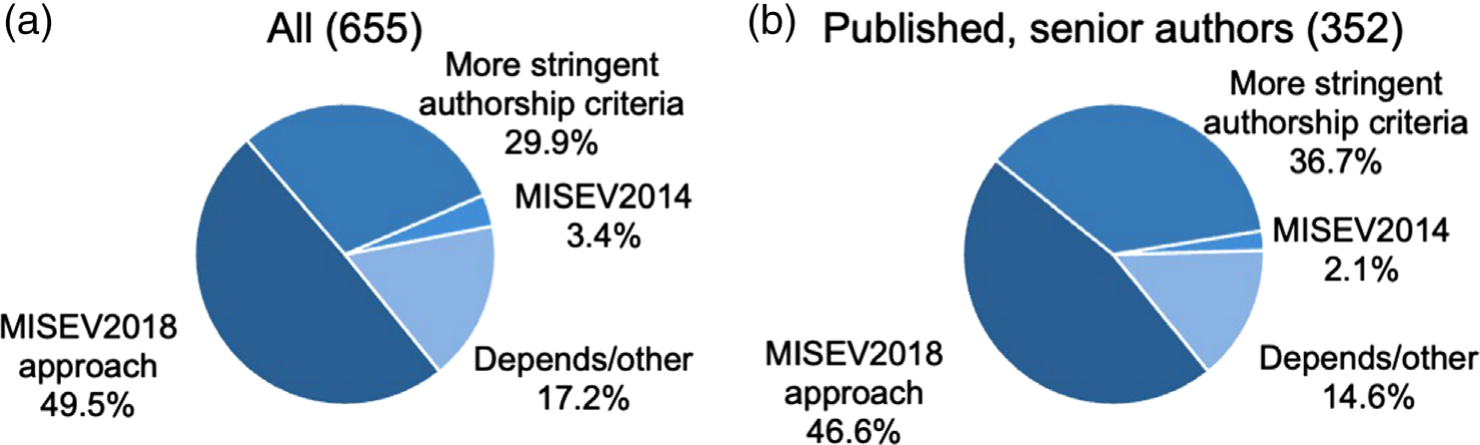
Preferences for authors of a MISEV update. Total number of respondents in each category is shown in parentheses in the panel titles

Almost 50% felt that the MISEV renewal should follow the same, broadly inclusive authorship procedures as MISEV2018, including all interested ISEV members and other invited experts (Figure 4a). However, 30% preferred more exclusive authorship criteria, such as requiring a terminal degree, a certain number of publications, or corresponding authorship on an EV publication. This percentage increased to nearly 37% if only senior members with at least one publication were considered (Figure 4b). Only around 3% of respondents preferred to return to the MISEV2014 model, in which only the ISEV Board of Directors and/or its designates would be authors. Almost 14% indicated that extent of authorship might be related to the size of the next MISEV product. In the free comments, one respondent suggested the “Delphi” method for consensus building. As for target date of publication, 76% preferred 2022, while 20% were willing to wait until 2023 or 2024.

**Conclusion**: A near‐majority of survey respondents favored the inclusive MISEV2018 model of authorship. However, a sizeable minority preferred more stringent authorship criteria, and this preference was stronger among more experienced EV researchers.

## 12 SPREADING THE WORD ON RIGOUR

A common perception about both of the prior MISEV efforts is that more visibility and outreach are needed. By December 2021, MISEV2018 had surpassed 3400 citations according to Google Scholar. However, during the same time, almost 14,000 related scholarly articles were listed in PubMed alone with EV‐related terms (e.g., [exosom* OR “extracellular vesicle” OR microvesicle OR ectosom*]). Indeed, this survey showed that even some ISEV members are not familiar with MISEV and in some cases had not previously heard of it. Five hundred sixty‐three scientists answered an optional question about whether and how they could help with outreach on current and future ISEV initiatives. Forty‐four percent were willing to “reach out personally to journal editors or associates, officials of regulatory bodies, or other organizations.” More than one third said they could “write and submit an editorial” or “mini‐MISEV” summary to a journal” in their subfield. Another third were willing to “advise ISEV on influential individuals and journals (…) who should be made aware of MISEV.” Around 28% said that they could not contribute or preferred not to be involved. One hundred thirty‐nine free‐form responses were recorded, many giving the names and contact information of editors, journals, and societies that could be approached in outreach efforts. Another idea that arose during Board discussions was to perform a detailed analysis of MISEV citations across journals and fields to identify outlets for focused follow‐up.

**Conclusion**: the current crowdsourcing effort harvested a wealth of information about outreach targets that should be exploited in parallel to a new MISEV update.

## 13 OVERALL CONCLUSIONS

Amidst continuing, massive growth in the EV field, efforts to enhance interpretability and comparability of results are more important than ever. Initiatives such as MISEV must evolve efficiently and be developed and presented as opportunities to strengthen EV research, rather than as barriers to innovation or entry into the field. Responses to a November 2020 survey on MISEV and other rigor efforts have revealed broad satisfaction with the shape and content of the most recent MISEV iteration, MISEV2018. However, survey takers also requested certain new emphases and additional information. In particular, the free‐form responses to several questions in the survey will provide the coordinating authors of the next MISEV manuscript with guidance for necessary changes to the framework and addition of new details and recommendations. As a final note on the survey results, fully 70% of survey respondents indicated a willingness to evaluate specific methods or reagents as needed in support of a MISEV update or other ISEV venture. This generosity and openness of EV researchers is commended and will ensure a successful new instalment of MISEV to guide the growth of EV research not just in quantity, but in quality as well.

## CONFLICTS OF INTEREST

EIB: Advisory board of Sphere Gene Therapeutics Inc.; JL: owns stock in Codiak BioSciences; ownership in Exocure biosciences Inc.; consults in the EV field through Vesiclebio AB; DCIG: Consultancy agreement with Codiak BioSciences.

## Supporting information

SUPPORTING INFORMATION

## References

[jev212182-bib-0001] Börger, V. , Weiss, D. J. , Anderson, J. D. , Borràs, F. E. , Bussolati, B. , Carter, D. R. F. , Dominici, M. , Falcón‐Pérez, J. M. , Gimona, M. , Hill, A. F. , Hoffman, A. M. , de Kleijn, D. , Levine, B. L. , Lim, R. , Lötvall, J. , Mitsialis, S. A. , Monguió‐Tortajada, M. , Muraca, M. , Nieuwland, R. , … Giebel, B. (2020). International Society for Extracellular Vesicles and International Society for Cell and Gene Therapy statement on extracellular vesicles from mesenchymal stromal cells and other cells: Considerations for potential therapeutic agents to suppress coronavir. Cytotherapy, 22(9), 482–485. 32425691 10.1016/j.jcyt.2020.05.002 PMC7229942

[jev212182-bib-0002] Clayton, A. , Boilard, E. , Buzas, E. I. , Cheng, L. , Falcón‐Perez, J. M. , Gardiner, C. , Gustafson, D. , Gualerzi, A. , Hendrix, A. , Hoffman, A. , Jones, J. , Lässer, C. , Lawson, C. , Lenassi, M. , Nazarenko, I. , O'Driscoll, L. , Pink, R. , Siljander, P. R. M. , Soekmadji, C. , … Nieuwland, R. (2019). Considerations towards a roadmap for collection, handling and storage of blood extracellular vesicles. Journal of Extracellular Vesicles [Internet], 2019 Dec 1 [cited 2020 Jul 28]; 8(1), 1647027. Available from: https://pubmed.ncbi.nlm.nih.gov/31489143/ 10.1080/20013078.2019.1647027 PMC6711123 31489143

[jev212182-bib-0003] Erdbrügger, U. , Blijdorp, C. J. , Bijnsdorp, I. V. , Borràs, F. E. , Burger, D. , Bussolati, B. , Byrd, J. B. , Clayton, A. , Dear, J. W. , Falcón‐Pérez, J. M. , Grange, C. , Hill, A. F. , Holthöfer, H. , Hoorn, E. J. , Jenster, G. , Jimenez, C. R. , Junker, K. , Klein, J. , Knepper, M. A. , … Martens‐Uzunova, E. S. (2021). Urinary extracellular vesicles: A position paper by the Urine Task Force of the International Society for Extracellular Vesicles. Journal of Extracellular Vesicles [Internet]. 2021 May 1 [cited 2021 Oct 3], 10(7), e12093. Available from: https://pubmed.ncbi.nlm.nih.gov/34035881/ 10.1002/jev2.12093 PMC8138533 34035881

[jev212182-bib-0004] Gardiner, C. , Vizio, D. D. , Sahoo, S. , Théry, C. , Witwer, K. W. , Wauben, M. , & Hill, A. F. (2016). Techniques used for the isolation and characterization of extracellular vesicles: Results of a worldwide survey. Journal of Extracellular Vesicles, 5(1), 32945. 27802845 10.3402/jev.v5.32945 PMC5090131

[jev212182-bib-0005] Lotvall, J. , Hill, A. F. , Hochberg, F. , Buzas, E. I. , Di Vizio, D. , Gardiner, C. , Gho, Y. S. , Kurochkin, I. V. , Mathivanan, S. , Quesenberry, P. , Sahoo, S. , Tahara, H. , Wauben, M. H. , Witwer, K. W. , & Théry, C. (2014). Minimal experimental requirements for definition of extracellular vesicles and their functions: A position statement from the International Society for Extracellular Vesicles. Journal of Extracellular Vesicles [Internet], 3, 26913. Available from: http://www.ncbi.nlm.nih.gov/pubmed/25536934 10.3402/jev.v3.26913 PMC4275645 25536934

[jev212182-bib-0006] Nieuwland, R. , Falcón‐Pérez, J. M. , Théry, C. , & Witwer, K. W. (2020). Rigor and standardization of extracellular vesicle research: Paving the road towards robustness. Journal of Extracellular Vesicles, 10(2), e12037. 33343835 10.1002/jev2.12037 PMC7735957

[jev212182-bib-0007] Poupardin, R. , Wolf, M. , & Strunk, D. (2021). Adherence to minimal experimental requirements for defining extracellular vesicles and their functions. Advanced Drug Delivery Reviews [Internet]. 2021 Sep 1 [cited 2021 Oct 3], 176, 113872. Available from: https://pubmed.ncbi.nlm.nih.gov/34284058/ 10.1016/j.addr.2021.113872 34284058

[jev212182-bib-0008] Royo, F. , Thery, C. , Falcon‐Perez, J. M. , Nieuwland, R. , & Witwer, K. W. (2020). Methods for separation and characterization of extracellular vesicles: Results of a Worldwide Survey Performed by the ISEV Rigor and Standardization Subcommittee. Cells, 9(9), 1955. 10.3390/cells9091955 PMC7563174 32854228

[jev212182-bib-0009] Théry, C. , Witwer, K. W. , Aikawa, E. , Alcaraz, M. J. , Anderson, J. D. , Andriantsitohaina, R. , Antoniou, A. , Arab, T. , Archer, F. , Atkin‐Smith, G. K. , Ayre, D. C. , Bach, J. M. , Bachurski, D. , Baharvand, H. , Balaj, L. , Baldacchino, S. , Bauer, N. N. , Baxter, A. A. , Bebawy, M. , … Zuba‐Surma, E. K. (2018). Minimal information for studies of extracellular vesicles 2018 (MISEV2018): A position statement of the International Society for Extracellular Vesicles and update of the MISEV2014 guidelines. Journal of Extracellular Vesicles, 7(1), 1535750. 30637094 10.1080/20013078.2018.1535750 PMC6322352

[jev212182-bib-0010] Van Deun, J. , Mestdagh, P. , Agostinis, P. , Akay, Ö. , Anand, S. , Anckaert, J. , Martinez, Z. A. , Baetens, T. , Beghein, E. , Bertier, L. , Berx, G. , Boere, J. , Boukouris, S. , Bremer, M. , Buschmann, D. , Byrd, J. B. , Casert, C. , Cheng, L. , Cmoch, A. , … Hendrix, A. (2017). EV‐TRACK: Transparent reporting and centralizing knowledge in extracellular vesicle research. Nature Methods, 14(3), 228–232. 28245209 10.1038/nmeth.4185

[jev212182-bib-0011] Welsh, J. A. , Van Der Pol, E. , Arkesteijn, G. J. A. , Bremer, M. , Brisson, A. , Coumans, F. , Dignat‐George, F. , Duggan, E. , Ghiran, I. , Giebel, B. , Görgens, A. , Hendrix, A. , Lacroix, R. , Lannigan, J. , Libregts, S. F. W. M. , Lozano‐Andrés, E. , Morales‐Kastresana, A. , Robert, S. , De Rond, L. , … Jones, J. C. (2020). MIFlowCyt‐EV: A framework for standardized reporting of extracellular vesicle flow cytometry experiments. Journal of Extracellular Vesicles [Internet]. 2020 Jan 1 [cited 2020 Jul 28], 9(1), 1713526. Available from: https://pubmed.ncbi.nlm.nih.gov/32128070/ 10.1080/20013078.2020.1713526 PMC7034442 32128070

[jev212182-bib-0012] Welsh, J. A. , van der Pol, E. , Bettin, B. A. , Carter, D. R. F. , Hendrix, A. , Lenassi, M. , Langlois, M. A. , Llorente, A. , van de Nes, A. S. , Nieuwland, R. , Tang, V. , Wang, L. , Witwer, K. W. , & Jones, J. C. (2020). Towards defining reference materials for measuring extracellular vesicle refractive index, epitope abundance, size and concentration [Internet]. Journal of Extracellular Vesicles, Taylor and Francis Ltd.; 2020 [cited 2020 Nov 30]. 9(1), 1816641. Available from: https://pubmed.ncbi.nlm.nih.gov/33062218/ 33062218 10.1080/20013078.2020.1816641 PMC7534292

[jev212182-bib-0013] Witwer, K. W. , Soekmadji, C. , Hill, A. F. , Wauben, M. H. , Buzás, E. I. , Di Vizio, D. , Falcon‐Perez, J. M. , Gardiner, C. , Hochberg, F. , Kurochkin, I. V. , Lötvall, J. , Mathivanan, S. , Nieuwland, R. , Sahoo, S. , Tahara, H. , Torrecilhas, A. C. , Weaver, A. M. , Yin, H. , Zheng, L. , … Théry, C. (2017). Updating the MISEV minimal requirements for extracellular vesicle studies: Building bridges to reproducibility. Journal of Extracellular Vesicles [Internet], 6(1), 1396823. Available from: https://www.tandfonline.com/doi/full/10.1080/20013078.2017.1396823 10.1080/20013078.2017.1396823 PMC5698937 29184626

