# Supplementary material for: Updating MISEV: Evolving the minimal requirements for studies of extracellular vesicles
Source: J Extracell Vesicles. 2021 Dec 25;10(14):e12182. doi: 10.1002/jev2.12182 (PMC8710080; doi:10.1002/jev2.12182)
Supplement: Supplementary file 1 — SUPPORTING INFORMATION [file JEV2-10-e12182-s001.pdf]

The "MISEV" minimal requirements were first published by the ISEV Board of Directors in 2014, followed in 2018 by a "MISEV2018" update with ISEV community involvement and nearly 400 co-authors. MISEV is not meant to be a final or static set of requirements, and periodic updates may be required, as is the case for recommendations in other rapidly developing scientific areas.

Please complete this 26-question survey to assist with evaluating the need for MISEV or related updates.

* 1. I am a:

- ◯ Senior investigator (more than four years since my terminal degree)
- ◯ Junior investigator (less than four years since my terminal degree or no terminal degree)

* 2. I study extracellular vesicles:

- ◯ As my major research focus
- ◯ As a large focus area, but I would consider myself more of an expert in other areas
- ◯ More incidentally to other research topics
- ◯ N/A: I don't currently study EVs but have done so in the past
- ◯ N/A: I don't study EVs but am interested in them and may work with them in future

* 3. I have studied EVs for:

- ◯ More than 10 years
- ◯ From 5 to 10 years
- ◯ Less than 5 years
- ◯ N/A. I do not study EVs

* 4. Regarding ISEV membership:

- ◯ I am currently an ISEV member and have been a member for at least five years total
- ◯ I am currently an ISEV member and have been a member for two to four years total
- ◯ I am currently an ISEV member and have been a member for less than two years
- ◯ I am not currently an ISEV member, but have been a member previously
- ◯ I am not and have not been an ISEV member

* 5. I have published this many primary research (reporting experimental data) manuscripts on EVs (can include preprints) :

- ◯ 0
- ◯ 1
- ◯ 2-5
- ◯ 6-10
- ◯ More than 10

* 6. I have read and am familiar with (check all that apply):

- ☐ The first MISEV publication (Lötvall et al, J Extracell Vesicles, 2014)
- ☐ The MISEV2018 update (Théry et al, J Extracell Vesicles, 2018)
- ☐ Neither MISEV publication

* 7. On MISEV2018:

- ◯ I am a co-author
- ◯ I contributed by completing one or more surveys, but am not a co-author
- ◯ I did not contribute and am not a co-author

* 8. On a possible future update to MISEV2018:

- ◯ I would like to contribute and be considered as a co-author
- ◯ I am glad to contribute via survey, but I prefer not to be a co-author
- ◯ I prefer not to contribute or be a co-author

Responses to MISEV2018

* 9. Since MISEV2018 appeared, I have (check all that apply):

- [ ] Urged adherence to MISEV2018 while reviewing articles or grant applications
- [ ] Mentioned MISEV2018 in a poster or oral presentation
- [ ] Encouraged lab members/colleagues to follow MISEV2018
- [ ] Promoted MISEV2018 or other rigor efforts of ISEV on social media
- [ ] Volunteered for or proposed an ISEV Rigor and Standardization task force
- [ ] N/A, none
- [ ] Other (please specify)

* 10. Regarding my publications in 2019 and 2020 ONLY (i.e. after the publication of MISEV2018), I:

- ○ Published on EVs and followed and/or cited MISEV2018
- ○ Published on EVs but did not follow or cite the MISEV2018 recommendations
- ○ Did not publish on EVs

* 11. The following most closely summarizes my views on MISEV and minimal requirements in the EV field:

- ○ Minimal requirements are important, and I support MISEV2018
- ○ Minimal requirements are important, but MISEV2018 is too restrictive
- ○ Minimal requirements are important, and MISEV2018 does not go far enough to uphold standards
- ○ Minimal requirements such as MISEV2018 are an unnecessary imposition on the EV field
- ○ I don't have strong feelings about minimal requirements or MISEV2018

12. In the OVERALL QUALITY of EV publications since 2018, I think that there has been:

- ◯ an improvement, and MISEV2018/other ISEV efforts have contributed to this
- ◯ an improvement, but not because of MISEV2018/other ISEV efforts
- ◯ no clear improvement or decline compared with previous years, perhaps because MISEV2018 is ineffective or its positive influence is balanced by a massive influx of low-quality studies
- ◯ a decline despite MISEV2018 (e.g., because MISEV2018 has not had sufficient uptake in the field or is known but not followed)
- ◯ Other (please specify)

13. In the quality of METHODS REPORTING in EV publications since 2018, I think that there has been:

- ◯ an improvement, and MISEV2018 and other rigor efforts, including the MISEV-endorsed EV-TRACK, have contributed to this
- ◯ an improvement, but not because of MISEV2018 and other rigor efforts
- ◯ no clear improvement or decline compared with previous years, perhaps because rigor efforts are ineffective or their positive influence is countered by a massive influx of low-quality studies
- ◯ a decline despite MISEV2018 and other rigor efforts (e.g., because MISEV2018 has not had sufficient uptake in the field or is known but not followed)
- ◯ Other (please specify)

* 14. I have received feedback from colleagues about MISEV2018 (checking all that apply):

- ☐ Praise for MISEV2018
- ☐ Endorsement of MISEV2018
- ☐ Complaints that MISEV2018 is too restrictive
- ☐ Complaints that MISEV2018 is too long
- ☐ Complaints that MISEV2018 left out important topics or details
- ☐ Suggestions that MISEV2018 has not had wide enough uptake
- ☐ Suggestions that MISEV2018 is not taken seriously
- ☐ None of the above or have not received feedback
- ☐ Other (please specify)

* 15. Any update to MISEV2018 should be:

- ○ Longer and more comprehensive, incorporating more topics and details
- ○ Around the same length and covering the same topics, but with updated details and references
- ○ Around the same length, but with at least partially new topical focus
- ○ Shorter, with fewer details and/or topics
- ○ Shorter, with fewer details, but accompanied by additional, detailed guidelines articles on specific topics (like EV sources, methods)
- ○ Other (please specify, but note that input on specific topics will be requested below in Q17)

* 16. Here are the main sections of MISEV2018. Which of these broad topics should be retained in a future update? Check all that apply. (If all should remain in the update, check them all.) Additional broad topics can be added in the comment box.

- ☐ Nomenclature
- ☐ Collection and pre-processing: pre-analytical variables
- ☐ EV separation and concentration
- ☐ EV characterization
- ☐ Functional studies
- ☐ Reporting requirements and exceptions
- ☐ Other section(s)?

* 17. Since MISEV2018, an ISEV Rigor and Standardization Subcommittee ("R&S") has been formed. R&S coordinates task forces on specific topics: sources of EVs (biofluids, culture medium, bacteria, tissue), EV reference materials, and regulatory affairs and EV therapeutics. Task forces are expected to make products such as reviews, recommendations, and position papers. How should task force products and MISEV relate to each other?

- ◯ The Rigor and Standardization task force products and MISEV updates are complementary and should be pursued separately and simultaneously
- ◯ The products of the Rigor and Standardization task forces should be integrated into any update to MISEV
- ◯ Immediate updates to MISEV may not be needed if the Rigor and Standardization task forces make topic-specific guidelines
- ◯ I don't know, would need more information, or don't have an opinion.
- ◯ Other (please specify)

18. Needed additions to MISEV include (select all that apply):

- ☐ More advice on pros and cons of different separation methods
- ☐ More advice on protein and non-protein markers to use for different types of EVs
- ☐ Advice on reliable antibodies for EV protein markers for different applications (WB, flow cytometry, immunohistochemistry, EM, etc.)
- ☐ Identification of topics for which task forces or other rigor efforts might be needed
- ☐ Other (please specify)

* 19. MISEV2018 and the Journal of Extracellular Vesicles endorse EV-TRACK (see www.evtrack.org) as a reporting tool to improve rigor and reproducibility.

- ◯ I support the endorsement of EV-TRACK and have used EV-TRACK myself/in my group
- ◯ I support the endorsement of EV-TRACK, although I have not used it myself/in my group
- ◯ I am not familiar with EV-TRACK
- ◯ I have no opinion on EV-TRACK
- ◯ I do not support the endorsement of EV-TRACK for the following reason(s):

20. Indicate any suggested changes to reporting requirements, including EV-TRACK. Please specify clearly WHICH reporting requirements are referred to.

* 21. My preferred approach to contributorship and authorship of a MISEV update is:

- The MISEV2018 approach should be repeated: The ISEV Board/designates will prepare a rough draft, which will be extensively reviewed and edited based on surveys. All current ISEV members will be invited to contribute via survey. Selected field leaders who may not be current ISEV members can also be invited by the ISEV Board.
- I generally agree with the MISEV2018 approach, but I feel that authorship should be more exclusive. Co-authors should be invited to contribute only if they have a certain degree experience in the field, such as a terminal degree, a minimum number of publications, or corresponding authorship on an EV publication.
- I do not agree with offering broad authorship for a MISEV update. In the interest of efficiency or for other reasons, senior contributors to the field, including the ISEV Board and its designates, should produce any update to MISEV2018. While input can be sought from the community on any and all points, authorship for contributors at this level is not necessary.
- The number/breadth of authors may depend of the form of the MISEV update. If long and exhaustive: wide authorship; if short and specific: authorship restricted to selected experts.
- Other (please specify)

* 22. If MISEV2018 is updated, we should aim to publish the new update:

- By or during 2022
- In 2023 or 2024
- Later than 2024
- I don't care, don't know, or prefer not to have an update

23. Understanding that the success of MISEV and any future updates depends on spreading the word, I am willing to (select all that apply):

- [ ] Reach out personally to journal editors or associates, officials of regulatory bodies, or other organizations, to share the current MISEV2018 guidelines and future updates and recommend that they be adopted (indicate below)
- [ ] Write and submit an editorial or "mini-MISEV" summary to a journal in my subfield on ISEV guidelines (give the name(s) below)
- [ ] Advise ISEV on influential individuals and journals in my field(s) who should be made aware of MISEV (provide details below)
- [ ] N/A. I cannot contribute or prefer not to be involved in outreach.
- [ ] Please indicate names of individuals, journals, organizations here, and give your name and email at the end of the survey if you would like to be contacted as part of this effort (optional)

* 24. If required, I/my laboratory am/are likely willing and able to devote a reasonable amount of resources to evaluate specific methods/reagents in the laboratory as needed to support a new MISEV release or other field-wide evaluation:

- ( ) Yes
- ( ) No

25. Comments not captured above?

26. Optional: My name and email address. (Completing this constitutes permission to be contacted.)

First (given) name

Last (family) name

email address
